# Mothers’ experiences and perceptions of breastfeeding peer support: a qualitative systematic review

**DOI:** 10.1186/s13006-024-00614-3

**Published:** 2024-01-19

**Authors:** Yuanyuan Yang, Huijuan Liu, Xiaoyi Cui, Jingwen Meng

**Affiliations:** 1 Peking University School of Nursing, Beijing, China; 2 Peking University Health Science Centre for Evidence-Based Nursing, Beijing, China; 3 Neonatal Disease Diagnosis and Treatment Center, Fifth Medical Center of Chinese People’s Liberation Army General Hospital, Beijing, China; 4 Peking University First Hospital, Beijing, China

**Keywords:** Breastfeeding, Peer support, Maternal, Experience, Perception

## Abstract

**Background:**

The global issue of low breastfeeding rates has been widely reported. Quantitative studies have shown the positive effects of peer support on breastfeeding. However, the experiences of mothers who receive breastfeeding peer support have been found to vary. To date, no systematic qualitative summary has been conducted to document the impact of peer support, nor to provide advice for its implementation from the perspective of breastfeeding mothers. This review aims to systematically synthesize qualitative findings on mothers’ experiences of breastfeeding peer support to provide evidence for optimizing peer support services and ultimately enhancing their role in promoting breastfeeding.

**Methods:**

PubMed, Embase, Cochrane Library, Ovid, Web of Science, CINAHL, China National Knowledge Infrastructure (CNKI), WanFang Datebase, VIP Database and Chinese Biomedical Database (CBM) were searched from the inception of each database until January 2023, to collect qualitative studies and mixed methods studies that included qualitative findings on mothers’ experiences with breastfeeding peer support. The Joanna Briggs Institute Qualitative Assessment and Review Instrument (JBI-QARI) was used to extract data and evaluate the quality of the included articles. The meta-integration method was used to explain and integrate the research findings. The review process was carried out by two authors independently, and the disagreements were resolved through consensus.

**Results:**

A total of 15 articles were included in the study, consisting of 13 qualitative studies and 2 mixed methods studies. The analysis identified four integrated themes: (1) obtaining psycho-emotional support; (2) acquiring knowledge and skills; (3) expectations for breastfeeding peer support; and (4) feeding perceptions and behavior change. It should be noted that the articles reviewed are in English and mostly originate from developed countries or regions. Therefore, the generalizability of the integrated findings to underdeveloped regions or non-English speaking countries may be limited.

**Conclusion:**

Mothers perceived that peer support had a positive impact on breastfeeding. To improve the effectiveness of peer support in promoting breastfeeding, it is important to consider the individual needs of each mother. It is recommended that peer support services should be standardized in the future, including the accreditation, training, supervision, and management of peer supporters.

**Supplementary Information:**

The online version contains supplementary material available at 10.1186/s13006-024-00614-3.

## Background

The World Health Organization (WHO) and UNICEF advocate that breastfeeding should continue until two years of age or longer due to significant benefits to both the mother and infant [1]. However, low breastfeeding rates are a global concern. According to the report released by WHO, the initiation rate of breastfeeding within one hour of birth was 47% in 2015–21, against the target of 70%. The exclusive breastfeeding rate at 6 months is 48%, falling short of the target of 70% by 2030. By two years of age, the breastfeeding rate drops to 45% [2]. Generally, breastfeeding duration is shorter in high-income countries than in low- and middle-income countries [3]. Therefore, efforts towards supporting breastfeeding must be amplified to reach the 2030 targets.

In recent years, the role of peer support in breastfeeding promotion has been recognized in several countries. Peer support isdefined as ‘the provision of emotional, appraisal, and informational assistance by a created social network member who possesses experiential knowledge of a specific behavior or stressor and similar characteristics as the target population [4]. Breastfeeding Peer Supporters, also known as Breastfeeding Peer Counselors (BPCs), are women with breastfeeding experience who volunteer to provide breastfeeding counseling to others in the community or hospital where they live [5]. Breastfeeding peer supporters are identified through selection or recruitment by health professionals, or through self-referral. Patterns of peer support vary widely around the world. Peer supporters also vary in the amount of training they receive.

There is evidence from quantitative studies that peer support is effective in supporting breastfeeding practices. A 2017 systematic review found that community-based peer support for mothers not only increased the duration of exclusive breastfeeding, particularly for infants aged three to six months in low- and middle-income countries, but also encouraged mothers to initiate breastfeeding early and prevented newborn prelacteal feeding of newborns [6]. A UK study found that one-to-one breastfeeding support provided by paid peer supporters and targeted at young mothers in the antenatal and postnatal periods was beneficial in increasing breastfeeding initiation and prevalence at two weeks [7]. Further studies have been conducted to explore what forms of peer support are feasible, acceptable, and effective. A home-based postnatal breastfeeding peer support programme delivered over six months in Hong Kong, China, was reported to be acceptable to women, but a more flexible approach to the number of visits and modification of the intensity of the intervention would be needed to increase retention [8]. In Australia, the RUBY trial conducted by Forster et al. demonstrated that the implementation and delivery of a proactive telephone breastfeeding peer support intervention was effective, feasible, sustainable and cost-effective [9, 10].

To better understand ways to optimally implement peer support, qualitative studies of mothers’ experience have been conducted. One study in Lebanon focused on peer support during antenatal breastfeeding education through home visits and phone calls [11], while a study in the UK focused on peer support through face-to-face coaching, phone calls, and text messages during both the antenatal and postnatal periods [12]. Mothers’ perceptions of the role of peer support varied between studies. Some studies found that peer support led to mothers perceiving that their knowledge about breastfeeding had improved [11, 12]; and Quinn’s study identified the impact of peer support on mothers’ social connectedness and the likelihood of mothers’ willingness to become breastfeeding advocates [13].

As can be seen, the timing, form, and role of peer support as well as mothers’ experience in the qualitative studies to date are diverse. There is a need to systematically review the current literature on mothers’ experiences of breastfeeding peer support. Despite two qualitative systematic reviews addressing breastfeeding peer support [14, 15], no updated reviews focus specifically on mothers’ experience and perspectives on peer supporters’ breastfeeding support. The information synthesized from this review could help to explain the successes and failures in implementing breastfeeding support from the mothers’ viewpoint. Furthermore, this allows a comprehensive understanding of how breastfeeding support offered by peers can be effectively carried out in the future. Therefore, the present review aimed to consolidate the qualitative evidence exploring mothers’ perceptions and experiences of the breastfeeding support they received from peer supporters.

## Methods

### Inclusion and exclusion criteria

The PICoS model [16] was used to construct the inclusion criteria: ① P (population): mothers who are breastfeeding; ② I (phenomenon of interest): mothers’ perceptions and experiences of breastfeeding peer support; ③ Co (context) specific scenario: providing mothers with breastfeeding peer support; ④ S (study design) type of research: qualitative study and mixed methods study. Exclusion criteria: ① literature with no access to full text; ② literature with low methodological quality; ③ literature not published in English or Chinese.

### Search strategy

Qualitative studies on mothers’ breastfeeding peer support experiences reported in PubMed, Embase, Cochrane Library, Ovid, Web of Science, CINAHL, China National Knowledge Infrastructure (CNKI), Wanfang database, VIP China Science and Technology Journal Database, and Chinese BioMedical Literature Database (CBM) were searched from inception to January 2023. Additionally, references to the included literature were manually searched for studies related to the topic. A combination of MeSH terms and free words was used to conduct the search. The search strategy was developed according to the specific requirements of different databases to collect relevant literature as comprehensively as possible. Search terms and structure in PubMed were presented in Table 1. The Chinese search terms corresponding to the above English search terms were used in the search of Chinese databases.

**Table 1:** Search terms and structure in PubMed

	Search terms and structure
1	peer support[Title/Abstract] OR peer-support[Title/Abstract] OR peer counselor[Title/Abstract] OR peer group[MeSH Terms] OR peer educator[Title/Abstract] OR peer mentor [Title/Abstract] OR peer advocate[Title/Abstract] OR volunteer[Title/Abstract] OR peer listener[Title/Abstract]
2	Breastfeeding [Title/Abstract] OR breastfed OR breast feeding[MeSH Terms] OR human milk[Title/Abstract] OR breast milk[Title/Abstract]
3	experience[Title/Abstract] OR feeling[Title/Abstract] OR expectation[Title/Abstract] OR perspectives[Title/Abstract]
4	qualitative research[MeSH Terms] OR interview[Title/Abstract] OR phenomenal study[Title/Abstract] OR grounded theory[MeSH Terms] OR focus group[MeSH Terms]
5	1 AND 2 AND 3 AND 4

### Literature screening and data extraction

Two researchers independently screened the literature, extracted the data from the literature, and then cross-checked it. Any disagreements were discussed until agreements were achieved. The articles were initially screened by reading the title and abstract and further screened by reading the full text to determine the final inclusion or not. An information extraction form was developed, and the data were extracted, including the authors, time of publication, study site, study methods, study population, phenomenon of interest, situational factors, and main findings. With regard to information that was lacking in the literature but essential for this review, the authors were contacted to obtain it if necessary.

### Methodological quality assessment

The JBI Critical Appraisal Checklist for Qualitative Research [16] was used for methodological quality assessment. Two researchers independently evaluated the quality of the included literature, and each item was scored as “yes”, “no” or “unclear”. The quality of the literature was graded as A if the evaluation items were all scored as “yes”; B if they were partially scored as “yes”’ and C if they were all not scored as “yes”. When disagreements occurred, discussion or consulting with a third party was conducted until a final agreement was reached. Only articles with quality grades A and B were included in this study.

### Data synthesis

Analysis of the included articles was conducted using the qualitative evidence synthesis method developed by JBI [16]. Each finding was read repeatedly, analyzed, and interpreted on the premise of critical appraisal. The findings with similar meaning were grouped to form new categories, and then the categories were pooled into syntheses. Subsequently, a comprehensive set of statements representing synthesized findings was generated. The qualitative evidence summation and synthesis were initially conducted by two researchers independently and then modified based on discussions with the entire research team.

## Results

**Fig. 1:**
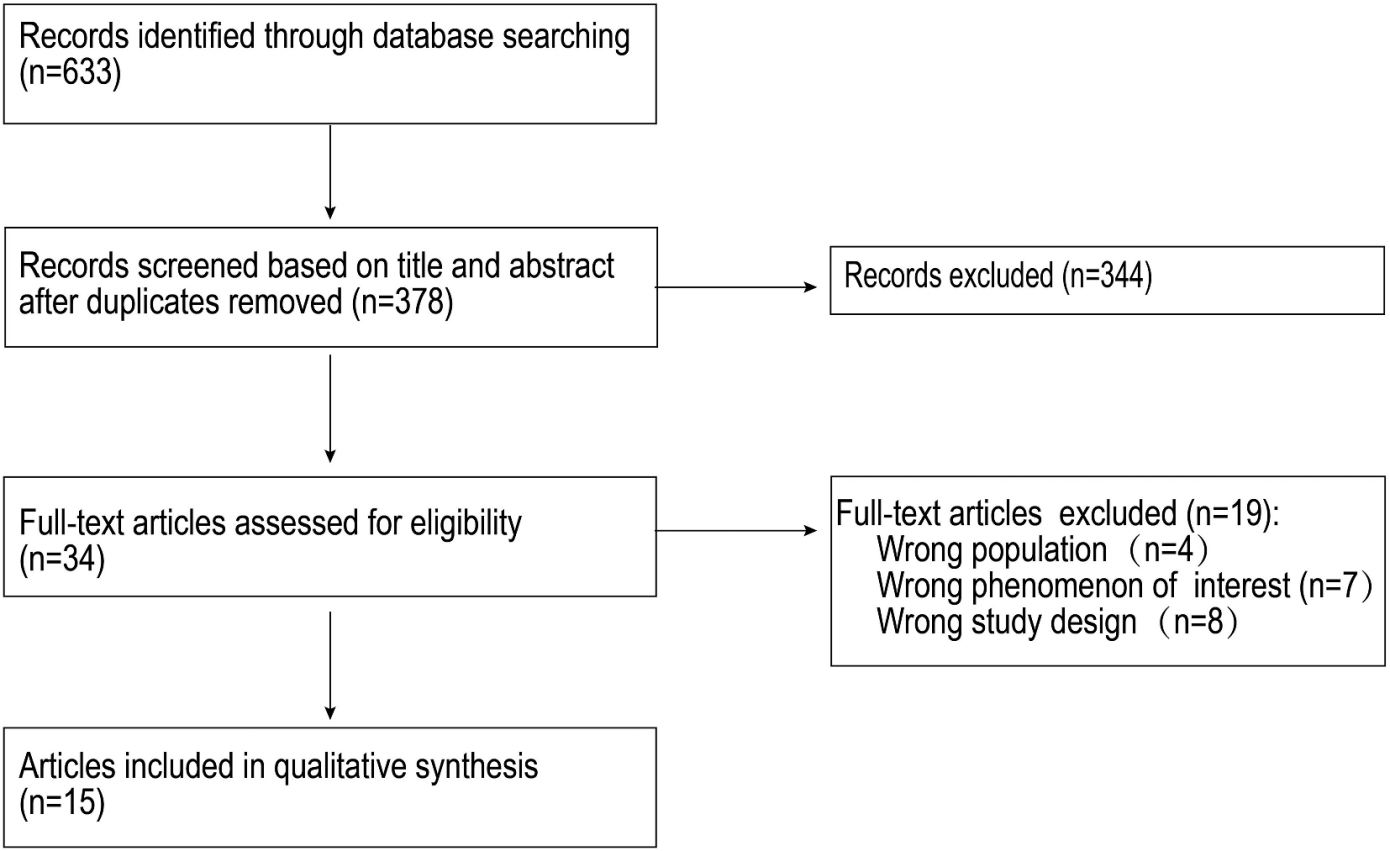
PRISMA flow diagram

The initial search yielded 633 articles. After removing duplicate records, 378 records were screened through titles and abstracts. Of those, 35 full-text articles were assessed for eligibility based on the inclusion criteria. Those not meeting the criteria were excluded, leaving 15 articles for this review (see Fig. 1 PRISMA flow diagram). The basic characteristics of the articles are shown in Table 2. Regarding the quality of the studies included, two [13,17] were graded as A, and 13 [8, 11, 12, 18–27] were graded as B (refer to Additional file 1).

**Table 2:** Characteristics of the included articles

First author (year of publication)	Country/district	Participants	Methodology	Phenomena of Interest	Setting/duration	Type of peer support	Findings
McLardie-Hore et al. (2022)^[18]^	Australia	10 mothers	Qualitative methods/Semi-structured in‑depth interview	Women’s experience of receiving proactive telephone-based peer support in a trial which was able to successfully increase breastfeeding maintenance at six-months	Home/20–65 min	Proactive telephone-based breastfeeding support from a trained peer volunteer for up to six months postpartum	Nonjudgmental support and guidance: nonjudgmental, compassionate and understanding positive experiences; practical advice; a social connection– more than just breastfeeding, not all support from family and friends is supportive
Clapton-Caputo et al. (2021)^[19]^	Australia	10 mothers	Qualitative descriptive approach/Semi-structured in‑depth interview	Mothers’ experiences and expectations of breastfeeding support in social media groups	Video chat or telephone/unstated duration	Social media support group	(1) Expecting and experiencing emotional support; (2) Receiving information to manage exclusive expressing
Lok et al. (2021)^[8]^	China/ Hongkong	5 mothers	Qualitative methods/In-depth interview	Mothers’ experiences of breastfeeding peer support	Telephone/Unstated duration	Five postnatal home-based visits with a trained volunteer peer supporter over a six month period	(1)Peer supporters provide positive knowledge and emotional support; (2)Peer support helps to increase confidence in breastfeeding; (3)Desire for peer support early and more contact via digital technologies
Black et al. (2020)^[20]^	UK/Northern Ireland	8 mothers	Exploratory qualitative approach/Semi-structured interview	Mothers’ experiences of breastfeeding support in a Facebook social media group	Public coffee shops/20–40 min	Social media breastfeeding support provided by volunteer peer supporters and moderated by administrators to ensure evidence-based information is being provided	(1)Increased breastfeeding self-efficacy; (2) Contributing factors: ‘education’, ‘accessibility’, ‘normalizing’, ‘extended goals’, and ‘online community’
Clarke et al. (2020)^[12]^	UK/England	30 mothers	Qualitative methods/ Interview	Mothers’ experience, acceptability, and satisfaction with breastfeeding peer support and perceived barriers or facilitators to effective delivery	Home/ 45–90 min	Proactive, woman-centred support using an assets-based approach provided by a paid peer support service or volunteers	(1) Care about the timing to receive the support; (2) Active support is valuable, prefer short message support; (3) Peer support provides social support and restructure the social environment
Ingram et al. (2020)^[21]^	UK/England	21 mothers	Qualitative methods /Semi-structured interview	Mothers’ views of the different components of breastfeeding peer support	Home/45–90 min	Proactive, woman-centred support using an assets-based approach provided by a paid peer support service or trained volunteers	(1) Early opportunities for infant feeding conversations/continuity of helper; (2) Mapping the friends and family tree; (3) Keeping in touch using proactive texting; (4) Knowing about local groups and assets; (5) Woman-centred approach
Kabakian-Khasholian et al. (2019)^[11]^	Lebanon	22 mothers	Cross-sectional, prospective, two group qualitative design/In-depth interview	Mothers’ experiences of breastfeeding peer support and the influence of the intervention on their social support system	Home/unstated duration	Postpartum peer telephone support provided by trained volunteering mothers	(1)Mothers valued the support from their peers and the IBCLCs. They appreciated the support provided by the IBCLCs much more than the peer support. (2) The main contribution of peer supporters as perceived by the mothers was the provision of moral support, which was perceived to be important in encouraging breastfeeding continuation; (3)Breastfeeding women did not enhance their social groups by including the peer supporters; (4)Breastfeeding mothers reached out to other mothers in their social circle.
Quinn et al. (2019)^[13]^	Ireland	15 mothers	Exploratory qualitative methods/Semi-structured interview	Mothers’ experiences of breastfeeding support groups	Online video/unstated duration	Volunteer breastfeeding support groups which are held in the community, are ongoing, with contact initiated by the mother, and run by trained breastfeeding counsellors	(1) Complexity of breastfeeding support; (2) Community and connection; (3) Impact of culture on breastfeeding needs; (4) The journey; (5) Passing on; (6) What mothers want.
Regan et al. (2019)^[22]^	UK/South Wales	14 mothers	Descriptive qualitative approach/Semi-structured interview	Mothers’ experiences of breastfeeding support in social media groups	Unstated setting/ 30–60 min	Online breastfeeding support: Facebook groups and forums where individuals engaged with each other	(1) Mothers were drawn to online support due to a lack of professional, familial, and partner support. (2) Benefits of online support: reassurance and normalizing, someone who has been through it, circle of peer support. (3) Limitations of online support: judgement, polarised debate; lack of regulation.
Robinson et al. (2019)^[17]^	America	22 African-American mothers	Prospective, cross-sectional qualitative study/Focus group	Experiences of African American mothers who participate in breastfeeding support groups on Facebook; mothers’ breastfeeding beliefs, decisions, and outcomes	Online video conferencing/ 60–90 min	Facebook group providing mom-to-mom breastfeeding support	(1) Creating a community for Black mothers; (2) Online interactions and levels of engagement; (3) Advantages of participating in online support groups, (4) Critiques of online support groups; (5) Empowerment of self and others, (6) Shifts in breastfeeding perceptions and decisions.
Robinson et al. (2016)^[23]^	America	9 African mothers	Qualitative methods/Focus group	Mothers’ breastfeeding experiences and the effect of breastfeeding peer counselors	Unstated	Peer support through clinic visits, telephone calls during the pregnancy and postpartum time periods, and in-hospital and in-home visits	(1) Educating with truth; (2) Validating for confidence; (3) Countering others’ negativity; (4)Supporting with solutions.
Ingram et al. (2013)^[24]^	UK/Bristol	163 mothers for survey and 14 mothers for interview	Concurrent triangulation mixed methods approach/On-line questionnaire survey and semi-structured interview	Mothers’ views of the targeted peer support service	Telephone and face-to-face interview/Understated	Targeted service of trained peer supporters providing antenatal and postnatal breastfeeding support	(1)Antenatal opportunity for knowledge; (2)Postnatal reassurance; (3)Encouragement and self-confidence; (4)Challenges of peer support–partners, building trust, role conflict.
Thomson et al. (2012)^[25]^	UK/north-west England	47 mothers	Qualitative exploratory approach/Focus group/Semi-structured in-depth interview	Mothers’ experiences, facilitators, barriers and challenges faced in the introduction of a breastfeeding peer support service	Face-to-face interviews were held at women’s homes/telephone interview/25–80 min	The Star Buddies service comprises trained paid and voluntary local breastfeeding mothers who provide antenatal/hospital and community breastfeeding peer support	(1) Providing realistic assessments across varying situational contexts; (2) Forming strategies and plans to help women overcome any obstacles; (3) Making women aware of any negative outcomes; (4) Mobilising external and personal resources to facilitate goal attainment; (5) Providing evaluations and feedback on women’s (and infants’) progress; (6) Helping women to focus their energy to achieve their breastfeeding goals.
Nankunda et al. (2010)^[26]^	Uganda	370 mothers	Mixed methods/Semi-structured interview	Women’s experiences of peer counselling for exclusive breastfeeding in an East African setting	Mothers’ homes/unstated duration	Individual peer counselling was offered to women, scheduled as five visits: before childbirth and during weeks 1, 4, 7 and 10 after childbirth. The trained peer counsellors were regularly supervised.	(1) Satisfaction with explanations by peer counsellors; (2) Spending enough time; (3) Usefulness of visits by peer counsellor, (4) Free interaction between peer counsellors and the women; (5) Future peer counselling is welcome.
Hoddinott et al. (2006)^[27]^	Scotland	206 women for survey/21 women for interview/8 groups for observation	Multi-method action research approach/Semi-structured interview/Observation/Focus group/Questionnaire survey	Women’s perceptions of one-to-one and group-based breastfeeding peer coaching and why groups were more popular	Interview held at women’s homes/Unstated duration	Group-based and one-to-one untrained peer coaching	Reasons for breastfeeding groups more popular than one-to-one coaching: socialising, normalising, and improving well-being by attending groups; visual experiences and breastfeeding during a group; diversity of communication in groups. Women often felt initial anxiety when attending a group for the first time, and they expressed doubt that one set of ‘‘breastfeeding rules’’ would suit everyone. One-to-one peer coaching was perceived as a greater risk to confidence and empowerment than group-based peer coaching.

**Fig. 2:**
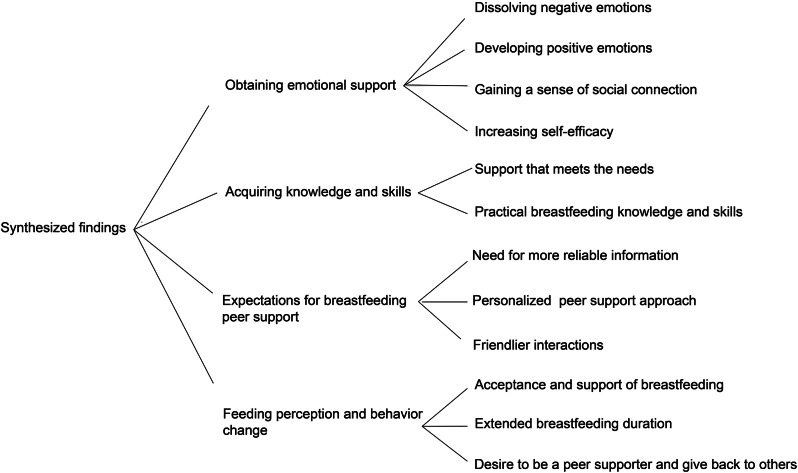
Synthesized findings

Through analysis of the 15 included articles, similar findings were grouped into 12 new categories and finally synthesized into 4 integrated findings (see Fig. 2 Synthesized findings).

### Synthesized findings 1: obtaining emotional support

Peer supporters provide emotional support to mothers, aiding them in reducing negative emotions, fostering positive emotions, feeling socially accepted, and gaining confidence in breastfeeding.

### Category 1: dissolving negative emotions

Peer support helps dissipate negative emotions mothers experience due to breastfeeding difficulties. The emotional support provided by peers with similar experiences allows mothers to feel less stress. Peer support also works for relieving mothers’ depression.

My failures in breastfeeding made me feel very frustrated and I had postpartum depression at that time, so I kept crying when [the volunteers] came to my home the first two times. They gave me emotional support [8].

I was so stressed because I couldn’t express and don’t know how to express.. and she helped me and to relax me.. [18].

Moreover, peer supporters helped mothers cope with the pain of grief.

She would phone me to make sure that everything was all right and I was not in any pain or anything and if I was worried about anything she would come and see me to check it.. because I was worried about a lot, so she came out a lot to see me. [25].

### Category 2: developing positive emotions

Peer support helps the mother keep calm and relaxed facing breastfeeding challenges.

She did a really useful thing actually, which was we did a map of people in my life that I could ask any help for feeding advice and things like that.. and just it just made me rethink and evaluate how much I appreciate having some family closer by. [12].

I was free with her because she’s a mother like me.” “.. The peer counsellor taught me during pregnancy and she came back after delivery when my breasts were very full, painful, and swollen, and then she helped me to express some breast milk and I felt relieved. [26].

Peers share their experiences, empathy, and encouragement enhancing the mother's sense of being understood and having positive feelings towards breastfeeding.

It’s just nice to speak to other women, and I’ve always felt more positive towards the feeding after going to the group. [21].

### Category 3: gaining a sense of social connection

Peer supporters are always friendly and warm. They share their personal experiences and emotions with each other, which draws an emotional connection between them. This closeness gives the mother a sense of social acceptance.

I think it was to meet other mums really and.. have a bit of one-to-one contact with other people. [13].

I think just having that additional person to talk to makes you feel less alone.. so it puts you at ease really about how you can actually do it. I think that’s essentially what you want, you want someone to have the same experience as you, you want someone to be like no it’s fine, you are okay. [12].

### Category 4: increasing self-efficacy

Peer supporters acknowledge and praise mothers for breastfeeding, which leads to positive feedback.

The counselor’s advice allowed me to be sure about my breastfeeding decision [23].

I always felt more positive about breastfeeding after attending the group. [22].

Mothers receive a self-efficacy boost with the help of their peers.

She [Peer supporter] gave me the confidence.. she passed me on an article, and I’d read that and we worked out things together... [13].

.. For me it was a really positive experience.. those calls and contacts through my first few months.. really gave me a lot of confidence to.. keep going.. [18].

Peer supporters share solutions for breastfeeding problems, and their experiences help mothers to believe in their ability to overcome difficulties and breastfeed successfully.

Very helpful to answer questions the midwives did not have time to go into; this makes a real difference in terms of motivation to continue breastfeeding. [24].

### Synthesized findings 2: acquiring knowledge and skills

Peer supporters provide mothers with breastfeeding knowledge and skills that are tailored to their needs and practical.

### Category 5: support that meets the needs

Peer supporters provide mothers with breastfeeding knowledge tailored to their needs.

Helped with my breastfeeding education.. that I needed. [23].

That’s what I need to know. Is it normal what he’s doing. I think if I’d found online groups and things earlier than I had, we might not have had so many tears in the first few weeks [22].

Peer supporters always know the effective way for mothers to be able to master breastfeeding knowledge and skills.

You could ask her questions and she’d explain them in a fashion that you could understand without being too medical.. and you could talk to her. [25].

.. I could understand what she taught me.. [26].

### Category 6: practical breastfeeding knowledge and skills

The advice and approaches offered by the peers were more practical because they had the advantage of having similar experiences.

I feel very strongly that this useful and practical advice given in the comfort of your own home environment in those very early days was an invaluable support. I can only believe that if more women were given this support there would be much more tendency to breastfeed [24].

It’s like you could go to your GP [General practitioner] and say I’ve got a screaming baby but actually they’re a male GP and they don’t really know. It’s not quite the same. It’s not that I don’t trust the doctor but you have a bit more faith in someone who’s been there, someone who’s been in the situation and can sympathise and say yeah, it’s not easy to breastfeed [22].

Peer supporters help mothers overcome feeding dilemmas with tips and techniques, and their guidance is hands-on.

.. The peer counsellor showed me how to put my baby on my breast properly and since it was the first experience for me, it was useful [26].

I have heard from the girls’ good tips, which I found work. It wasn’t just one tip, they gave me a range of different tips for maybe over one problem [27].

... I had some lumps in my breasts…and they were really, really sore.. um and I had an idea that they were.. some sort of blockage.. she did offer some very good advice for getting in the shower and.. So it never progressed any further than that... [18].

### Synthesized findings 3: expectations for breastfeeding peer support

Mothers expect reliable breastfeeding support through friendly interactions with peer supporters. There are variations in their preference for peer support.

### Category 7: need for more reliable information

Peer supporters generally do not have a medical background, so the information provided by them is not always perceived by mothers as completely reliable.

It is about your personal experience, just talking to other people in the same group.. There is no reliable information there. [13].

In particular, it was considered difficult to effectively identify information on digital platforms.

I have lingering doubts in my mind, especially about issues that I am concerned about, and I always think about the accuracy of some information [obtained on the internet]. [21].

### Category 8: personalized peer support approach

Mothers had varied expectations for peer support, with some preferring digital technology, including phone calls, text messages, and social service platforms. Many mothers said they liked texting for peer support and communication..

... Text message was better because at that point I was always feeding him, so it was quite difficult to get the phone, so with the text it was more easy because I just answered when I could and she the same. [12].

Some mothers used to obtain peer support through online social media. Social media provides quicker and more timely access to information than face-to-face communication.

... Such as FaceTime, which is convenient for volunteers because they don’t have to do home visits, and also good for mothers; otherwise, the mothers have to arrange a time to meet [volunteers], which will be stressful. [8].

When I had nipple thrush, I definitely searched the internet for nipple thrush and read what people said about help for that. [13].

Facebook helps find the content of interest and offers help in a tailored way. [19].

Peer support in the form of groups can help mothers receive more rich input from a variety of different people.

People have different requests, but other people have given answers.. which is truly niche. [13].

I think it is great to have professionals involved as well. [20].

Because of different cultural backgrounds, mothers need support from specific breastfeeding peers with similar experiences, and they need support from dedicated media groups even more..

Well, we have many experiences that are unique to Black women that we cannot explain to other people, so an inclusive group is definitely needed. [17].

However, some mothers felt that it was still face-to-face peer support that allowed them to receive more beneficial help.

She calls you, but that is not enough to benefit from her. It is no better than someone who spends an hour and a half visiting you and teaching you baby care. [22].

Regarding the timing of peer support, some mothers wanted help early to receive longer and more frequent interactions to help them cope with any problems that might arise.

If I were well prepared before birth, it would be much better than preparing after birth. [8].

In contrast, some mothers preferred later.

I did not really want to acknowledge until the 20-week scan.. 12-weeks.. I do not think I was even thinking about post-birth. [12].

### Category 9: friendlier interactions

Mothers felt that social platforms, due to their anonymity or nonface-to-face nature, led to more direct or carefree expressions from those online, leading to some negative emotional experiences for mothers. They would like to see friendlier discussions on social platforms.

I think sometimes conversations can get a little heated. And um, I think they’re a little harsh at times for people who may be, are not using the search feature or asking a very commonly, a common question and people just kind a pounce on them. So, I think people may get discouraged. [17].

You would want to get honest opinions from people, not criticism or something like that. [13].

I just got into a heated argument with someone I have never met before, and I do not think that would happen in real life. [22].

### Synthesized findings 4: feeding perception and behavior change

Peer support has a significant impact on mothers’ perceptions and behaviors towards breastfeeding. It encourages them to accept and adhere to breastfeeding, and even inspires them to become breastfeeding peer supporters themselves.

### Category 10: acceptance and support of breastfeeding

Peer support helps mothers accept breastfeeding by addressing misconceptions about breastfeeding.

... It’s sort of give me confidence to not really care about the rest of societies [society’s] opinions as long as I am happy with my own mothering and parenting. [22].

Breastfeeding images allow mothers to accept breastfeeding in public.

I feel the images are amazing. They’re so uplifting. There was one, um, that empowered me so much the other day. It was a woman breastfeeding her daughter at a restaurant, and today, I thought about that, and I’ve been thinking about it for days, and I went out today, and I did it... [17].

The support the mother gained from the peers reinforces her to continue breastfeeding.

Well originally before I came across the Breastfeeding in Northern Ireland page I think originally my goal was to get to the minimum six-month period and now having educated myself my goal is probably to get to either the age of two or a natural weaning point or when I get pregnant again and I can’t... [20]

If I didn’t have the peer supporter to talk to about things I would be more likely to give up [24].

Mothers who received peer support were more likely to be breastfeeding advocates.

I think also it’s nice to be able to give support, because in these groups you’ve got people who have just had babies and it’s nice to be able to say actually I’ve been there now. So, where people have answered my questions, I can hopefully answer theirs [22].

#### Category 11: extended breastfeeding duration

Peer support increases mothers’ confidence to achieve their goal of breastfeeding longer.

Without this study I could have stopped breastfeeding from the first month.. Without the help of the peer support and consultant [11].

It’s been great to see that there’s women that do it for 12 months, two years, and so that six-month goal doesn’t seem so unachievable. It kind of almost makes you feel– it motivates you to keep going. [19].

### Category 12: desire to be a peer supporter and give back to others

As mothers became more familiar with breastfeeding with the help of peer support, they became very willing to offer help to others.

Because it’s important to see women that look like us doing this stuff. Marrying that up with what I learned in the class that I took for the CLC [Certified Lactation Consultant]. So, yeah, this [breastfeeding] would have been a done deal, and I wouldn’t be this advocate. I wouldn’t be posting breastfeeding on my Facebook page, I wouldn’t be going to support groups if it wasn’t for the group. [17].

Providing support to other mothers not only gave them a sense of accomplishment but also enhanced their own breastfeeding experience.

... My need to empower other women with up to date, correct information is being met.. making new friends hopefully, and.. feeling the breastfeeding love.. [13].

## Discussion

This synthesis of findings on mothers’ perceptions and experiences of breastfeeding peer support through a meta-integration approach confirms the values of peer support, identifies ways to optimize the effect of peer support, and provided administrators with recommendations for the effective operation of peer support. Evidence from the included studies revealed that breastfeeding peer support has multiple positive effects for mothers. Peer supporters are empathetic to the mother’s breastfeeding experience because of their own breastfeeding experience, and can therefore provide them with emotional support that meets their needs and helps them overcome negative feelings such as loneliness, sadness, and depression during breastfeeding. In addition, peer support can improve mothers’ self-efficacy in achieving breastfeeding goals, and therefore help them to breastfeed more actively. Peers can provide mothers with more relevant and desired knowledge, although they are not professional healthcare providers. It is concluded that breastfeeding peer support is a worthwhile initiative to promote breastfeeding.

Most mothers wanted higher frequency, longer duration, and more interactive peer support. However, there was a wide variation in preference for forms of peer support. Some mothers felt that one-to-one face-to-face peer support could lead to a mutually supportive and decision-making experience [11], while others indicated that group-based peer support might be a better option because of the anxiety felt in one-to-one support [20, 27]. Additionally, there were variations as to the peer support needed by mothers from different cultural backgrounds. African-American mothers preferred to be supported by teams composed of Black mothers because they felt that only Black mothers could understand their experiences [17]. Because the challenges mothers face during breastfeeding are always complex and the needs for peer supporters are dynamic, peer support teams from diverse backgrounds are better able to meet the mothers’ needs. Therefore, individualized peer support should be provided to mothers according to their needs and background.

Variability in the quality and competence of peer supporters leads to different experiences of peer support for mothers. Quality peer supporters are more likely to have a positive effect on mothers’ breastfeeding experience [17]. Peer supporters face transitional difficulties as they move from a voluntary to a professionalized role [28]. This suggests the need for professional training for peer supporters to respond to the needs of mothers. Training plays an important role in improving the attitudes of peer supporters as well as enhancing their knowledge [29]. Peers who have scientific knowledge and skills in breastfeeding are more likely to gain the trust of mothers and provide a good experience for them. Therefore, standardized training standards for breastfeeding peer support should be developed for peer support to be more effective.

With the development of digital technology, mothers are able to quickly access peer support from social networks with similar experiences. However, mothers do not always have the ability to recognize reliable information online [22]. They need reliable knowledge from healthcare professionals. It is suggested that a social platform for breastfeeding support can be built with the participation of health professionals [20]. Peers with similar experiences communicate and interact with mothers on this platform to provide peer support to mothers, and health professionals can provide timely supervision of the services of peer supporters to ensure reliable support.

Although currently, most of the peer supporters are volunteers, the peer support services require adequate funding for recruitment, training, and ongoing supervision of peer supporters, as well as paying them travel expenses. In addition, peer supporters, although their motivations to become a peer supporter are ‘give something back’ to the service that had supported them, some of them still expect to be paid. Limited funding causes a high turnover because they need to get back to paid employment. In the UK, peer support services are facing a reduction in available funding [29]. This impacted on the quality of peer support services. Accordingly, further investment must be made to establish effective breastfeeding peer support services.

There is also a need to facilitate the interface between breastfeeding peer support agencies and mothers. Effective communication methods can help mothers understand the usefulness of peer support and enable them to be willing to try the positive effects of peer support [26]. Mothers who receive peer support often show a greater willingness to provide peer support if they have a good experience, which further expands the number of peer supporters and is more conducive to sustainability [13, 17].

In addition, research has shown that many mothers look forward to peer support from social media groups [19, 20, 22]. They often turned to online breastfeeding support due to a lack of face-to-face support [30]. From this perspective, online support provides a service that is of great need. Due to the time- and space-independent properties of online support, women have convenient access to online support [19]. Moreover, online support allows for large-scale outreach in a short period of time, quickly establishing a connection between peers and mothers. With the prevalence of mobile health, the online breastfeeding support may alleviate the financial burden for governments, and thus provide a cost-effective way to increase breastfeeding rates [20]. Therefore, the use of online platforms should be explored to provide a sustainable peer support model in the future.

### Limitations

Since the literature included in this study was in English and mostly originated from developed countries or regions, the applicability of the integrated results to underdeveloped regions or non-English speaking countries was weak. Whereas, the fact that the studies came from different countries with different health care systems and cultural norms related to breastfeeding may also be seen as a strength. Moreover, gray literature was not searched and included, which may have led to an incomplete inclusion of literature. Additionally, this qualitative integration used a descriptive methodology which provides limited interpretation of meaning, but the method is applicable to studies that explore perspectives and experiences.

### Implications for research and practice

First, most of the included studies were conducted in developed countries, indicating that mothers’ experience on breastfeeding peer support is less studied in underdeveloped regions, or that there are fewer relevant studies conducted in these regions published in English. Consequently, studies focusing on this topic are encouraged in these areas to enrich the recognition of breastfeeding peer support. Additionally, relevant studies exploring feasible and effective forms of breastfeeding peer support are also recommended from the perspectives of different stakeholders. Second, adequate funding should be provided to facilitate the breastfeeding peer support. Ongoing training mechanisms and dynamic peer support monitoring are recommended, in order to fully exert the role of peer support in promoting the breastfeeding.

## Conclusion

This study systematically evaluated mothers’ experiences of breastfeeding peer support. The results confirmed that mothers perceived positive effects of peer support on breastfeeding. It is important to note that the peer support analyzed in the included studies varied in terms of the characteristics of the peer supporters and the training they received, as well as the form and timing of the support. Therefore, it is recommended that peer support services should be standardized, including the accreditation, training, supervision, and management of peer supporters in the future. Additionally, exploring measures to promote the sustainability of peer support is necessary. The review’s findings also suggest that individual needs of mothers should be assessed before providing peer support. Personalized and targeted peer support should be provided based on the individual’s preferences and willingness.

### Electronic supplementary material

Below is the link to the electronic supplementary material.

**Supplementary file 1: Table 3.** Results of quality appraisal using the JBI Critical Appraisal Checklist

## Data Availability

All data generated or analyzed during this study are contained within the manuscript.

## Abbreviations

BPC: Breastfeeding peer counselors
CBM: Chinese BioMedical Literature Database
CNKI: China National Knowledge Infrastructure
JBI–QARI: Joanna Briggs Institute–Qualitative Assessment and Review Instrument
WHO: World Health Organization
