# Supplementary material for: Mothers’ experiences and perceptions of breastfeeding peer support: a qualitative systematic review
Source: Int Breastfeed J. 2024 Jan 19;19:7. doi: 10.1186/s13006-024-00614-3 (PMC10797811; doi:10.1186/s13006-024-00614-3)
Supplement: Supplementary file 1 — Table 3. Results of quality appraisal using the JBI Critical Appraisal Checklist [file 13006_2024_614_MOESM1_ESM.docx]

Table 3 Results of quality appraisal using the JBI Critical Appraisal Checklist

| Author(s) and Year | 1 | 2 | 3 | 4 | 5 | 6 | 7 | 8 | 9 | 10 | Grade |
| --- | --- | --- | --- | --- | --- | --- | --- | --- | --- | --- | --- |
| McLardie-Hore et al. (2022)^[18]^ | Unclear | Yes | Yes | Yes | Yes | No | Yes | Yes | Yes | Yes | B |
| Clapton-Caputo et al. (2021)^[19]^ | Unclear | Yes | Yes | Yes | Yes | Yes | Yes | Yes | Yes | Yes | B |
| Lok et al.(2021) ^[8]^ | Unclear | Yes | Yes | Yes | Yes | No | No | Yes | Yes | Yes | B |
| Black et al. (2020)^[20]^ | Yes | Yes | Yes | Yes | Yes | No | Yes | Yes | Yes | Yes | B |
| Clarke et al. (2020)^[12]^ | Unclear | Yes | Yes | Yes | Yes | Yes | No | Yes | Yes | Yes | B |
| Ingram et al. (2020) ^[21]^ | Unclear | Yes | Yes | Yes | Yes | Yes | No | Yes | Yes | Yes | B |
| Kabakian-Khasholian et al. (2019)^[11]^ | Unclear | Yes | Yes | Yes | Yes | Yes | Yes | Yes | Yes | Yes | B |
| Quinn et al. (2019)^[13]^ | Yes | Yes | Yes | Yes | Yes | Yes | Yes | Yes | Yes | Yes | A |
| Regan et al. (2019)^[22]^ | Unclear | Yes | Yes | Yes | Yes | No | No | Yes | Yes | Yes | B |
| Robinson et al.(2019) ^[17]^ | Yes | Yes | Yes | Yes | Yes | Yes | Yes | Yes | Yes | Yes | A |
| Robinson et al. (2016)^[23]^ | Unclear | Yes | Yes | Yes | Yes | No | No | Yes | Yes | Yes | B |
| Ingram et al.(2013) ^[24]^ | Unclear | Yes | Yes | Yes | Yes | No | No | Yes | Yes | Yes | B |
| Thomson et al. (2012)^[25]^ | Yes | Yes | Yes | Yes | Yes | No | No | Yes | Yes | Yes | B |
| Nankunda et al. (2010)^[26]^ | Unclear | Yes | Yes | Yes | Yes | No | Yes | Yes | Yes | Yes | B |
| Hoddinott et al. (2006)^[27]^ | Unclear | Yes | Yes | Yes | Yes | No | No | Yes | Yes | Yes | B |
| Note: 1. Is there congruity between the stated philosophical perspective and the research methodology? 2. Is there congruity  between the research methodology and the research question or objectives? 3. Is there congruity between the research  methodology and the methods used to collect data? 4. Is there congruity between the research methodology and the representation and analysis of data? 5. Is there congruity between the research methodology and the interpretation of results? 6. Is there a statement locating the researcher culturally or theoretically? 7. Is the influence of the researcher on the research, and vice-versa, addressed? 8. Are participants, and their voices, adequately represented? 9.Is the research ethical according to current criteria or, for recent studies, and is there evidence of ethical approval by an appropriate body? 10. Do the conclusions drawn in the research report flow from the analysis, or interpretation, of the data? | | | | | | | | | | | |
